# Gentamicin Adsorption onto Soil Particles Prevents Overall Short-Term Effects on the Soil Microbiome and Resistome

**DOI:** 10.3390/antibiotics10020191

**Published:** 2021-02-15

**Authors:** Concepcion Sanchez-Cid, Alexandre Guironnet, Laure Wiest, Emmanuelle Vulliet, Timothy M. Vogel

**Affiliations:** 1 Environmental Microbial Genomics, Laboratoire Ampère, UMR 5005, CNRS, Ecole Centrale de Lyon, Université de Lyon, 69134 Ecully, France; vogel@univ-lyon1.fr; 2 Promega France, 69100 Charbonnières-les-Bains, France; 3 Institut des Sciences Analytiques, Université Claude Bernard Lyon 1, CNRS, Université de Lyon, 69100 Villeurbanne, France; Alexandre.GUIRONNET@isa-lyon.fr (A.G.); Laure.WIEST@isa-lyon.fr (L.W.); Emmanuelle.VULLIET@isa-lyon.fr (E.V.)

**Keywords:** soil resistome, antibiotic adsorption, antibiotic pollution, bioavailability, gentamicin, soil metagenomics

## Abstract

Antibiotics used in agriculture may reach the environment and stimulate the development and dissemination of antibiotic resistance in the soil microbiome. However, the scope of this phenomenon and the link to soil properties needs to be elucidated. This study compared the short-term effects of a range of gentamicin concentrations on the microbiome and resistome of bacterial enrichments and microcosms of an agricultural soil using a metagenomic approach. Gentamicin impact on bacterial biomass was roughly estimated by the number of 16SrRNA gene copies. In addition, the soil microbiome and resistome response to gentamicin pollution was evaluated by 16SrRNA gene and metagenomic sequencing, respectively. Finally, gentamicin bioavailability in soil was determined. While gentamicin pollution at the scale of µg/g strongly influenced the bacterial communities in soil enrichments, concentrations up to 1 mg/g were strongly adsorbed onto soil particles and did not cause significant changes in the microbiome and resistome of soil microcosms. This study demonstrates the differences between the response of bacterial communities to antibiotic pollution in enriched media and in their environmental matrix, and exposes the limitations of culture-based studies in antibiotic-resistance surveillance. Furthermore, establishing links between the effects of antibiotic pollution and soil properties is needed.

## 1. Introduction

Among all the ecosystems present on Earth, soil harbors the highest microbial diversity [1] and is likely the biggest reservoir of antibiotics. Most of the antibiotics currently used in human therapy and food production have been isolated from soil bacteria and fungi [2]. A natural consequence of this production of antibiotics by soil bacteria is the development of antibiotic resistance in soil. Soil is considered as one of the main environmental reservoirs of antibiotic-resistance genes (ARGs) [3]. Both clinically relevant and novel ARGs have been identified even in low-anthropogenically impacted soils [4,5,6,7], showing that antibiotic resistance occurs in soil even in the absence of a strong anthropogenic selective pressure. Furthermore, antibiotic resistance genes present in soil are often associated with mobile genetic elements (MGEs) [8] and, therefore, can be transferred to both other environmental bacteria and human pathogens [9].

The use of antibiotics in agriculture and the application of manure from antibiotic-treated animals for soil fertilization increase antibiotic selective pressure and ARG-containing microorganisms in the environment [10,11]. This selective pressure may enhance the development of antibiotic resistance in soil and its mobilization and transfer to clinically relevant bacteria. Therefore, the risks associated with the use of antibiotics in agriculture must be evaluated in order to regulate their use. Multiple studies over the last two decades have analyzed the effects of antibiotic-polluted manure composting on the soil microbiome. They have shown that this manure can increase the abundance of antibiotic-resistant bacteria (ARB), ARGs and MGEs [12,13,14,15,16,17]. However, soil is a highly complex and diverse matrix that changes over time [18]. Several factors, such as soil characteristics (i.e., water content, oxygen concentration or nutrient availability) [19,20], the percentage of reduced bioavailability of antibiotics due to their adsorption onto soil [21,22] and the activity and resilience of soil bacteria [23,24] may alter the effects of antibiotics in soil. In addition, soil is a solid matrix, and the physical contact between bacteria and antibiotic compound is reduced in comparison to liquid environments. These differences may inhibit accurate evaluation of the horizontal transfer of ARGs between resistant and susceptible bacteria in soil. Soil characteristics can provide a strong buffering capacity for antibiotic pollution [25] by reducing the impact of antibiotics on the soil microbiome and the potential consequences of soil pollution with antibiotics on human health. Thus, a global picture of the effects of antibiotic pollution on resistance development in soil is difficult to determine, and after decades of research, the scope of this phenomenon still remains unclear [26].

This study compared the response of soil bacteria to antibiotic pollution in soil microcosms and enrichments in selective media. Gentamicin is an aminoglycoside used in both human therapy and food production, and genes conferring resistance to gentamicin are widely distributed in the environment [27]. A range of concentrations of gentamicin from 1 µg/g of soil up to 1 mg/g of soil were added to an agricultural soil with no previous known exposure to gentamicin to evaluate their effects on the soil microbiome and resistome. Bacterial enrichments were contaminated with gentamicin up to 12 µg/mL. The main hypothesis was that gentamicin would be strongly adsorbed onto soil particles and that gentamicin pollution has a higher effect on soil bacteria in enriched media than in soil microcosms, since it would be more bioavailable, and bacteria in enriched media are more responsive to gentamicin, given their higher activity and lower diversity, and the higher availability of nutrients. This work demonstrates the differences in the microbiome and resistome response to gentamicin pollution between soil microcosms and bacterial enrichments, and exposes the limitation of culture-based studies in antibiotic-resistance surveillance in terrestrial ecosystems.

## 2. Results

### 2.1. Gentamicin Effect on Soil Bacterial Communities

**Figure 1:**
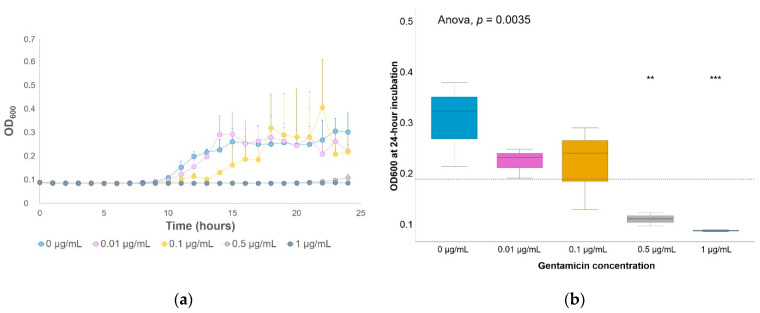
Gentamicin growth inhibition on soil bacterial enrichments. Optical density measured at 600 nm (OD_600_) of cultivable soil bacteria incubated in 1:10 tryptic soy broth (TSB) with different gentamicin concentrations: (**a**) for every hour during a 24-h incubation at 29 °C; (**b**) after 24-h incubation. Significant differences between each group (each gentamicin concentration) and the average between all groups (horizontal dashed line) were determined by a t-test. ** *p*-value ≤ 0.01; *** *p*-value ≤ 0.001. *n* = 3.

The highest tested concentration of gentamicin that could be considered as subinhibitory for enriched soil bacteria at a cursory level was 0.1 µg/mL since it did not cause significant overall growth inhibition, and the growth curve approached that of the nonpolluted soil bacteria enrichments (Figure 1). On the other hand, a delay of 15 h on the offset of the curve was observed at 0.5 µg/mL of gentamicin, and there was no visible growth in soil bacteria enrichments with 1 µg/mL of gentamicin (Figure 1a). In addition, significantly lower optical densities were measured at 600 nm (OD_600_) in bacterial enrichments with 0.5 and 1 µg/mL of gentamicin when compared to the average of all samples (Figure 1b). Both concentrations were considered, therefore, to be inhibitory for enriched soil bacteria. The inhibitory concentration selected for samples undergoing DNA extraction was scaled up to 12 µg/mL of gentamicin to ensure that inhibitory effects were observed. DNA was extracted from bacterial liquid enrichments with 0.1 µg/mL of gentamicin (a subinhibitory concentration), 12 µg/mL of gentamicin (an inhibitory concentration) and in control enrichments with no added gentamicin.

**Figure 2:**
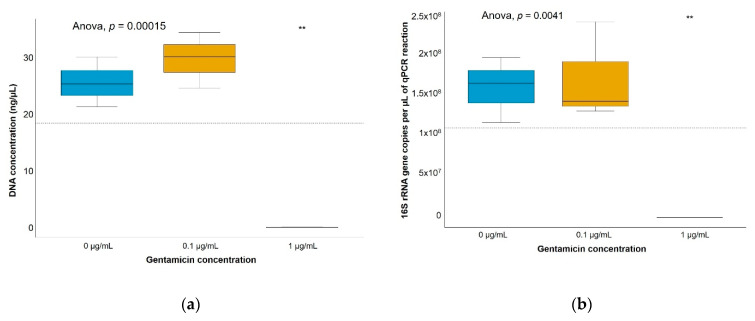
Gentamicin effect on soil bacterial growth in 1:10 TSB medium enrichments after 24-h exposure. (**a**) DNA concentrations and (**b**) number of 16S rRNA gene copies/µL of quantitative-PCR (qPCR) reaction. Significant differences between each group (each gentamicin concentration) and the average between all groups (horizontal dashed line) were determined by a t-test. ** *p*-value ≤ 0.01. *n* = 3.

**Figure 3:**
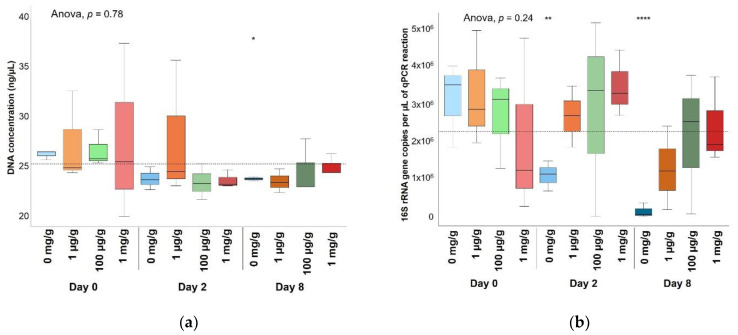
Bacterial dynamics over time in soil microcosms at different gentamicin concentrations. (**a**) DNA concentrations and (**b**) number of 16S rRNA gene copies/µL of qPCR reaction obtained from nonpolluted soil microcosms or microcosms polluted with gentamicin at 1 µg/g, 100 µg/g or 1 mg/g. Significant differences between each group (each concentration and each exposure time) and the average between all groups (horizontal line) were determined by a t-test. * *p*-value ≤ 0.05. ** *p*-value ≤ 0.01. **** *p*-value ≤ 0.0001. *n* = 3.

Significantly lower DNA concentrations and biomass were detected in soil bacterial enrichments with 12 µg/mL of gentamicin (Figure 2), showing a clear inhibitory effect, whereas gentamicin concentrations of up to 1 mg/g did not show a significant decrease of DNA concentrations nor bacterial biomass in soil microcosms (Figure 3). Non-gentamicin soil microcosms showed a significantly lower number of 16S rRNA gene copies than soil with gentamicin after both a 2-day exposure and an 8-day exposure. Non-gentamicin soil microcosms also showed a significantly lower DNA concentration than soil with gentamicin after an 8-day exposure. The significant differences found between soil microcosms with and without added gentamicin may reflect, rather than a growth stimulation induced by gentamicin, small differences in the community composition between soil triplicates polluted at the same gentamicin concentration and exposure times. The differences in the bacterial size of gentamicin-polluted soil microcosms may also account for handling error during DNA extraction and quantitative-PCR (qPCR) amplification. Thus, no obvious effect of gentamicin on the size of the bacterial community was observed.

**Figure 4:**
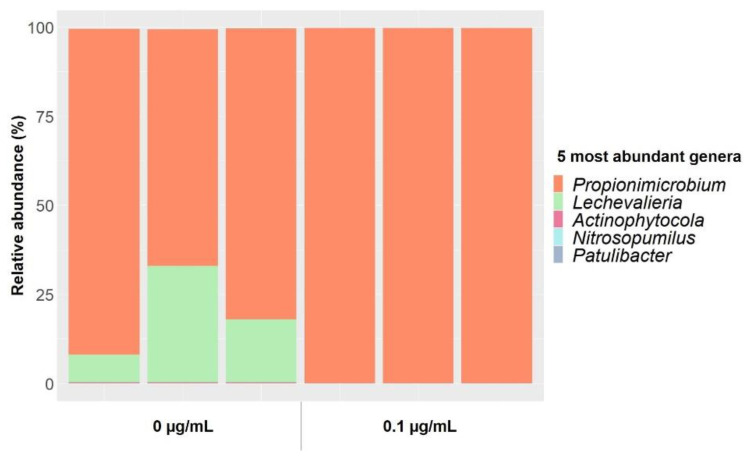
Relative abundances of the five most abundant genera from soil bacteria enrichments in 1:10 TSB medium incubated at 29 °C for 24 h at gentamicin concentrations of 0 and 0.1 µg/mL. Triplicates are plotted individually.

Five genera represented almost 100% of the total communities extracted from soil bacteria enrichments in 1:10 tryptic soy broth (TSB) medium (Figure 4). *Propinimicrobium* was the most prevalent genus, representing between 65 and 90% of the total in non-gentamicin (control) enrichments and almost 100% in enrichments at gentamicin concentrations of 0.1 µg/mL. Enrichments at 12 µg/mL of gentamicin could not be sequenced, since DNA concentrations were very low and the 16S rRNA gene could not be amplified. Whereas the composition of the bacterial communities in non-polluted enrichments varied between triplicates, gentamicin at 0.1 µg/mL reduced those differences (Figure S1a). In addition, a significantly lower genus richness was detected in gentamicin-contaminated enrichments when compared to noncontaminated controls (Figure S2). These results suggest that gentamicin, even at subinhibitory concentrations, inhibits some members of the community in bacterial enrichments.

**Figure 5:**
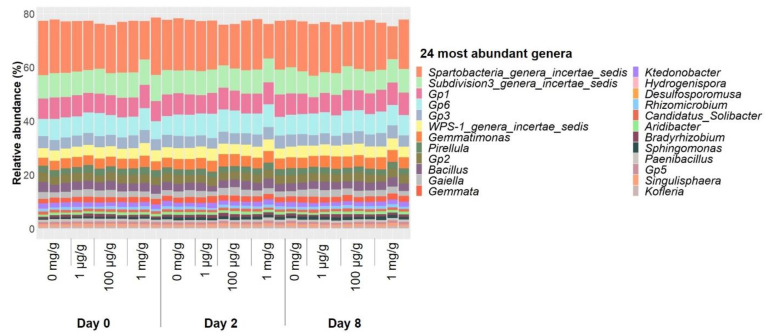
Relative abundances of the 24 most abundant genera in the total communities from nonpolluted soil microcosms or microcosms with 1 µg/g, 100 µg/g or 1 mg/g of gentamicin. Triplicates are plotted individually.

On the other hand, the relative abundance of the 24 most abundant genera in soil microcosms experienced only little changes under gentamicin pollution over time (Figure 5), and both the overall composition of the bacterial communities (Figure S1b) and genus richness (Figure S2) remained stable after 8-day exposure. None of the gentamicin concentrations used in this study had an overall inhibitory effect on soil microcosms, and even concentrations at mg/g levels showed little effect on the composition of soil bacterial communities after 8-day exposure, while 12 µg/mL of gentamicin had clear inhibitory effects on the culturable fraction of the soil microbiome. This may partly be due to differences in the communities present in soil microcosms and bacterial enrichments (Figure S1c). The genus richness measured in bacterial enrichments was significantly lower than that of most of the communities present in soil microcosms (Figure S2). Therefore, the proportion of culturable soil bacteria did not represent the diversity present in soil microcosms.

### 2.2. Gentamicin Effect on the Soil Resistome

**Figure 6:**
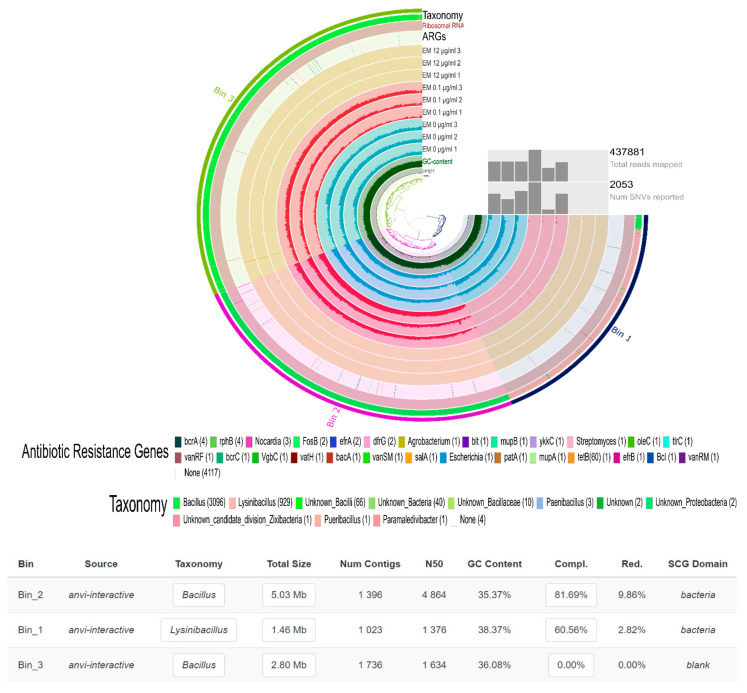
Assembly of metagenomic samples obtained from soil bacterial enrichments. EM: enriched medium; 1,2,3: triplicates from each condition.

Regarding soil bacterial enrichments, samples at 12 µg/mL were generally absent in the metagenomic assembly, while samples from nonpolluted enrichments and enrichments polluted at 0.1 µg/mL of gentamicin showed a high alignment rate (Figure 6). Two metagenome-assembled genomes (MAGs) were obtained from the assembly. The first one had a completion of 81.69% and a redundancy of 9.86%, and belonged to the genus *Bacillus.* The second one had a completion of 60.56% and a redundancy of 2.82%, and belonged to the genus *Lysinbacillus.* Both MAGs were virtually absent in samples at 12 µg/mL of gentamicin, and their presence did not show any obvious link to gentamicin. Many contigs (39) contained different (27) ARGs (Figure 6). However, none of these genes were specific to gentamicin. On the other hand, only 137 contigs were coassembled from soil microcosms, and the maximum alignment rate obtained when mapping metagenomic reads against the contigs was 0.08%. No ARGs were identified when blasting the contigs against the CARD database and filtering at 60% identity and 33 amino acid length.

**Figure 7:**
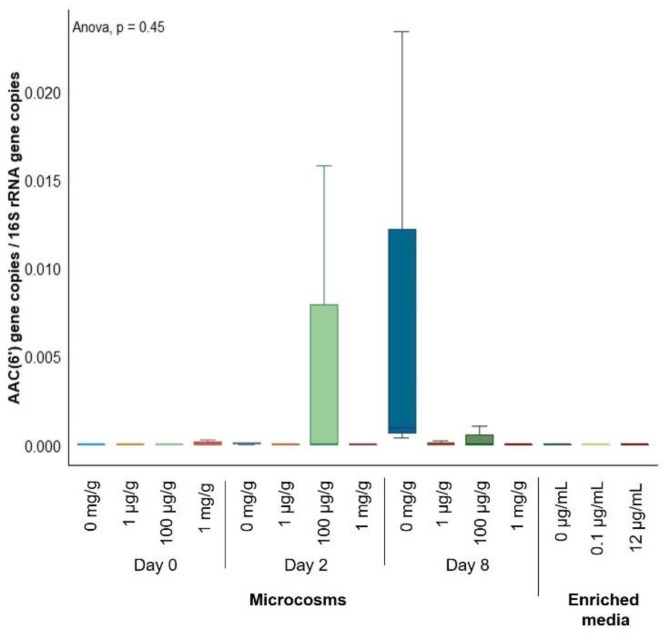
Dynamics of the abundance of gentamicin-resistance genes in soil microcosms and enriched media with and without gentamicin. Number of copies of gentamicin-resistance genes from the AAC(6′) aminoglycoside acetyltransferase family per µL of qPCR reaction normalized by the number of copies of the 16S rRNA gene per µL of qPCR reaction.No significant differences were found between conditions (each gentamicin concentration and each exposure time for soil microcosms and each gentamicin concentration after 24-h exposure for bacterial enrichments). *n* = 3.

Two genes that encode for components of multidrug efflux pumps, *muxB* and *adeF,* were found to significantly increase their relative abundance in nonassembled metagenomic sequences from bacterial enrichments polluted at 12 µg/mL of gentamicin (Table 1). However, none of these efflux pumps were related to gentamicin efflux. Thus, although gentamicin causes significant effects on the bacterial communities of soil bacterial enrichments, a selection for genes conferring resistance to gentamicin was not obvious. Regarding soil microcosms, 26 genes showed an increased relative abundance over exposure time in nonassembled metagenomic sequences from gentamicin-polluted soils (Table S1). Of these genes, 5 were related to aminoglycoside resistance, 11 were related to multidrug efflux, 5 to tetracycline efflux and 5 to other mechanisms. However, when applying the leave-one-out cross-validation to each of these genes, none of these increases in relative abundance were found to be significant. In addition, although almost all samples from soil microcosms and noncontaminated bacterial enrichments contained genes coding for enzymes of the AAC(6′) aminoglycoside acetyltransferase family that had been overlooked by metagenomic sequencing, these were low-abundant, and a significant increase on their abundance with gentamicin present was not detected (Figure 7). Therefore, gentamicin, even at high concentrations on the order of mg/g, did not induce any significant change in the resistome of soil bacteria.

The available fraction of gentamicin in soils polluted at the concentrations used in this study was measured by high-performance liquid chromatography/mass spectrometry (HPLC-MS/MS) to estimate the percentage of gentamicin that was adsorbed onto soil particles (Table S2). No trace of gentamicin was detected in any of the measured samples, suggesting that it was almost completely adsorbed onto soil particles. Although a small percentage of gentamicin can escape this adsorption, it was under the quantification limit (10 ng/mL) of the technique used in this study and below the inhibition threshold determined with enriched media. Thus, even when high concentrations of gentamicin were added to soil, the bioavailable fraction was subinhibitory. The lack of bioavailability of gentamicin in soil partially accounts for the differences observed between the response of soil bacteria in microcosms and enriched media, where gentamicin was not adsorbed onto soil particles and had an immediate effect on soil bacteria.

## 3. Discussion

The main goal of this study was to compare the response of soil bacteria to the antibiotic gentamicin in soil microcosms and enriched media using a metagenomic approach. The effects of gentamicin pollution on the soil microbiome and resistome were analyzed at three levels: overall effects on bacterial growth, impact on the community composition and potential selection for ARGs. As hypothesized, clear differences were observed between the response to gentamicin of soil bacteria enriched in selective media and in soil microcosms. These differences probably lay in the limited bacterial diversity present in bacterial enrichments and in gentamicin adsorption onto soil particles.

The reduced richness detected in bacterial enrichments (Figure S2) and the differences observed in bacterial composition between soil microcosms and bacterial enrichments (Figure S1c) support the concerns about the use of culture-based studies for antibiotic surveillance in terrestrial ecosystems. The majority of the bacteria present in soil are uncultured [28,29], and these uncultured soil bacteria are a reservoir of ARGs [6]. In addition, in complex environments such as soil, other selective forces such as nutrient availability or predation are likely to take place [30]. The impact of the environmental and bacterial interactions on the development of antibiotic resistance is critical and should not be overlooked [31,32,33]. Therefore, these results demonstrate the need to analyze the effects of antibiotics using microcosms and field studies when possible, since the response observed using culture-based approaches does not necessarily reflect what happens in the environment.

In addition, this research illustrates how the effects of the same antibiotic are strongly dependent on the environmental matrix. Concentrations that were overall subinhibitory in bacterial enrichments perturbed the bacterial community structure without changing their resistome. Concentrations that were roughly four orders of magnitude higher than the inhibition threshold did not cause significant short-term effects on soil bacteria in their environmental matrix due to gentamicin adsorption onto soil particles. Thus, this study demonstrated the need to take environmental physico-chemical properties into account in antibiotic-surveillance studies in terrestrial ecosystems and to systematize antibiotic-concentration measurement, since both antibiotic structure and soil properties affect the behavior of antibiotics in the receiving environment [34]. Furthermore, these results demonstrated the limitations of the terms “subinhibitory” and “inhibitory” in complex environments. These terms were initially defined in single cultures, and they may not be descriptive of the dose-response relationships taking place in complex communities in situ [35], where some members of the community may be inhibited even at concentrations below the overall inhibitory threshold.

Finally, gentamicin-resistance genes were detected by qPCR (Figure 7), even though they had been undetected during metagenomic analyses. Although they provide a more complete version of the environmental microbiome than culture-based experiments, metagenomic techniques are still biased and do not uncover all soil bacterial diversity [36]. Sequencing technologies such as the one used in this study (MiSeq sequencing) may not sequence deeply enough to obtain MAGs from samples as rich and diverse as soil. Deeper sequencing technologies should be used in this kind of matrix in order to obtain a more accurate picture of the soil microbiome [37] and identify the bacterial hosts of environmental ARGs.

Different antibiotic–soil combinations may cause different effects on the soil microbiome. However, this study evaluated the short-term effects of gentamicin after a single dose. Since antibiotic sequestration is a reversible process [11], the bioavailability of gentamicin could increase at longer exposure times and cause long-term perturbations on the soil microbial communities. In addition, several studies have shown that environmental factors, such as particle size or mineral composition, create specific microenvironments adequate for the growth of specific bacterial taxa [38,39,40]. This generates a spatial heterogeneity in the soil microbial communities at a small scale. Moreover, antibiotic concentrations in the environment likely form gradients in soil, and these gradients are likely associated with soil resistome heterogeneity at a small scale. The different populations in soil may respond differently to the same antibiotic according to their physiological state [41]. Thus, the dilution of local heterogeneity during DNA extraction at higher sample sizes might hide changes at a microscale. Further studies should analyze the effects of antibiotics in soil microenvironments and account for differences related to soil spatial heterogeneity, since studies designed to observe general changes in soil microcosms after antibiotic addition might overlook any event happening at a local scale and, therefore, underestimate the risk associated with antibiotics in soil.

## 4. Materials and Methods

### 4.1. Soil Sampling

Soil was sampled from a plowed corn field at La Côte de Saint André, France (45°38′ N–5°26′ E) on January 2018, on which manure from farm animals treated with cefalexin, neomycin, cefalonium, tetracycline, oxytetracycline, tylosin and sulfamidine was applied. This soil had no previous exposure to gentamicin. Soil characteristics are described in Table 2. Ninety-six sampling points were randomly selected within the field, and soil was shoveled at 20 cm depth. Five kg of sample were mixed together and kept at 4 °C until the start of the experiments.

### 4.2. Microcosm Experiment

Soil from La Côte de Saint André stored at 4 °C was sifted at 4 mm and homogenized, and 100 g microcosms without vegetation were prepared in polypropylene containers, since gentamicin has shown to be highly adsorbed onto glass [42]. Soil water retention capacity was 24.2% ± 0.64%. Microcosms were left at room temperature overnight before pollution with 1 µg/g, 100 µg/g or 1 mg/g of gentamicin (Duchefa Biochemie, Haarlem, The Netherlands). Serial dilutions of gentamicin were made in water, and 1 mL of solution was applied to soil four times intermittently, mixing with a metal bar in between applications. Triplicates were made for each concentration, as well as for non-polluted samples. DNA was extracted after 0-, 2- and 8-day incubation at ambient temperature and light without moisture maintenance treatment.

### 4.3. Determination of Gentamicin Effect on Soil Enrichments

First, in order to determine whether gentamicin had an effect on the enriched fraction of soil bacteria in liquid enrichments and which gentamicin concentrations were subinhibitory at a cursory level, soil bacteria were extracted in 0.9% NaCl solution, and 150 µl of 1:10 tryptic soy broth (TSB) medium containing soil bacteria without antibiotics or with gentamicin at 0, 0.01, 0.05, 0.1, 0.5 or 11 µg/mL were transferred to a 96-well culture plate and incubated for 24 h at 29 °C under continuous shaking in the MultiSkan GO Plate Reader (Thermo Scientific, Waltham, MA, USA). Optical density at 600 nm (OD_600_) was measured every hour over the 24-h incubation. Then, ANOVA tests and Student’s t-tests between the OD_600_ measured at each gentamicin concentration after 24-h incubation and the average between all groups were performed using the ggpubr package in R.

### 4.4. Enrichment of Soil Bacteria in 1:10 TSB Medium Polluted with Gentamicin

Soil bacteria were extracted in 0.9% NaCl solution, and 0.5 mL of extracted bacteria were added to 4.5 mL of 1:10 TSB medium without antibiotics or polluted with gentamicin at 0.1 and 12 µg/mL. Enrichments were incubated at 29 °C for 24 h. Then, 1 mL of soil bacteria enrichments was centrifuged at 2000× *g* for 5 min. The pellet was resuspended in 100 µL of 10 mg/mL lysozyme and 400 µL of TE buffer. After heating at 37 °C for 30 min shaking at 800 rpm, the lysate was purified using the Maxwell RSC Instrument and the Maxwell RSC Whole Blood DNA Kit (Promega, Madison, WI, USA).

### 4.5. DNA Extraction from Soil Microcosms

DNA was extracted using the Maxwell RSC Instrument (Promega) and a prototype version of the Maxwell Fecal Microbiome Kit (Promega), and 250 mg of sample were diluted in 1 mL of lysis buffer (Promega) and heated for 5 min at 95 °C. Samples underwent bead-beating twice at 5.5 m/s for 30 s in Lysing Matrix E tubes (MP Biomedicals, Irvine, CA, USA) and centrifuged at 10,600× *g* for 5 min. Then, 300 µL of supernatant was added to 300 µL of binding buffer (Promega) and loaded into a Maxwell RSC cartridge containing magnetic beads for DNA purification on the Maxwell RSC Instrument, according to Technical Manual TM473.

### 4.6. DNA Quantification and Quantitative PCR (qPCR) Assays

DNA concentrations extracted from soil microcosms and bacterial enrichments were assessed using the Qubit Fluorometer and the Qubit dsDNA HS Assay Kit (Thermo Fisher). The size of the total bacterial community was estimated by quantifying the V3 region of the 16S rRNA gene by qPCR using the “universal” primers 341F (5′-CCT ACG GGA GGC AGC AG- 3′) and 534R (5′-ATT ACC GCG GCT GCT GGC A-3′) [43,44]. A primer pair (F: 5′-CATGACCTTGCGATGCTCTATG-3′; R: 5′-TCCAAGAGCAACGTACGACTG-3′) was designed to target a 201-bp conserved region of 15 genes belonging to the AAC(6′) acetyltransferase family: *aac(6′)-Ib, aac(6′)-Ib’, aac(6′)-Ib-cr, aac(6′)-Ib3, aac(6′)-Ib4, aac(6′)-Ib7, aac(6′)-Ib8, aac(6′)-Ib9, aac(6′)-Ib10, aac(6′)-Ib11, aac(6′)-30/aac(6′)-Ib’, aac(6′)-Ib-Hangzhou, aac(6′)-Ib-Suzhou, aac(3)-Ib/aac(6′)-IIb”, ant(3”)-II/aac(6′)-IId.* The qPCR assays were carried out using the Corbett Rotor-Gene 6000 (QIAGEN) in a 20 µL reaction volume containing GoTaq qPCR Master Mix (Promega), 0.75 µM of each primer and 2 µL of DNA at ≤2.5 ng/µL. Two nontemplate controls were also included in all the assays. Standard curves for 16S rRNA gene assays were obtained using 10-fold serial dilutions of a linearized plasmid pGEM-T Easy Vector (10^7^ to 10^2^ copies) containing the 16S rRNA gene of *Pseudomonas aeruginosa* PAO1. Cycling conditions for 16S rRNA gene qPCR amplification were 95 °C for 2 min followed by 30 cycles of 95 °C for 15 s, 60 °C for 30 s and 72 °C for 30 s. Standard curves for *aac(6′)* gene qPCR assays were obtained from river water DNA, cloned and transformed using the TOPO TA cloning Kit (Invitrogen), linearized and diluted (10^7^–10^2^ copies/µL). Cycling conditions for qPCR amplification were 95 °C for 2 min followed by 35 cycles of 95 °C for 30 s, 55 °C for 30 s and 72 °C for 30 s. Melting curves were generated after amplification by increasing the temperature from 60 °C to 95 °C. The number of copies per µL of reaction obtained from the amplification of *aac(6′)* genes was normalized by the copies of the 16S rRNA gene per µL of reaction to assess their relative abundance in soil microcosms and bacterial enrichments. Then, ANOVA tests and Student’s t-tests between the DNA concentration, copies of 16S rRNA gene per µl of reaction and relative abundance of *aac(6′)* genes measured from each group (each gentamicin concentration after 24-h exposure for bacterial enrichments and each gentamicin concentration and each exposure time for microcosms) and the average between all groups were carried out using the ggpubr package in R.

### 4.7. 16S rRNA Gene and cDNA Sequencing and Analysis

The V3-V4 hypervariable region of bacterial 16S rRNA gene weas amplified from DNA obtained from both enriched bacteria and soil microcosms except for enriched media polluted at 12 µg/mL due to insufficient DNA concentration. DNA was amplified using the Titanium Taq DNA Polymerase (Takara Clontech, Kyoto, Japan) forward 341F with Illumina overhang (5′-TCG TCG GCA GCG TCA GAT GTG TAT AAG AGA CAG TCG TCG GCA GCG TCA GAT GTG TAT AAG AGA CAG CCT ACG GGN GGC WGC AG-3′) and reverse 785F with Illumina overhang (5′-GTC TCG TGG GCT CGG AGA TGT GTA TAA GAG ACA GGT CTC GTG GGC TCG GAG ATG TGT ATA AGA GAC AGG ACT ACH VGG GTA TCT AAT CC-3′) primers [45]. Amplification conditions were as follows: 95 °C for 3 min followed by 25 cycles of 95 °C for 30 s, 55 °C for 30 s and 72 °C for 30 s, and a final extension step at 72 °C for 5 min. DNA libraries were prepared from amplified products based on Illumina’s 16S Metagenomics Library Prep Guide (15044223 Rev. B) using the Platinum Taq DNA Polymerase (Invitrogen, Carlsbad, CA, USA) and the Nextera XT Index Kit V2 (Illumina, San Diego, CA, USA). DNA sequencing with a 15% PhiX spike-in was performed using the MiSeq System and the MiSeq Reagent Kit v2 (Illumina). Reads were trimmed to meet a quality score of Q20. Then, pair-ended reads were assembled using PANDAseq [46] at a sequence length between 410 and 500 bp and an overlap length between 20 and 100 bp, using the rdp_mle algorithm. Finally, each of the DNA sequences was annotated to the genus level using the Ribosome Data Project (RDP) database and the RDP Bayesian classifier using an assignment confidence cut-off of 0.6 [47]. Three microcosm samples were excluded from further analyses due to insufficient sequencing depth: a triplicate polluted at 1 µg/g of gentamicin after 0-day exposure, a triplicate polluted at 1 µg/g of gentamicin after 2-day exposure and a non-polluted triplicate after 8-day exposure. The genera that had less than 10 associated sequences in the ensemble of sequences from enriched media and soil microcosms were removed. Then, the relative abundances of the remaining genera were calculated and plotted individually. PCoA analyses were performed using R to compare the community composition of soil microcosms and bacterial enrichments. In addition, the genus richness measured in each sample was determined using the vegan package in R [48]. Then, ANOVA tests and Student’s t-tests between the genus richness detected in each group (each gentamicin concentration after 24-h exposure for bacterial enrichments and each gentamicin concentration and each exposure time for microcosms) and the average between all groups were carried out using the ggpubr package in R.

### 4.8. Metagenomic Sequencing and Analysis

Metagenomic libraries were prepared from <1 ng of DNA obtained from both enriched bacteria and soil microcosms using the Nextera XT Library Prep Kit and Indexes (Illumina), as detailed in Illumina’s Nextera XT DNA Library Prep Kit reference guide (15031942 v03). DNA sequencing with a 1% PhiX spike-in was performed using the MiSeq System and the MiSeq Reagent Kit v2 (Illumina). Two approaches were used to evaluate the antibiotic resistome of nonpolluted and gentamicin-polluted soil bacterial enrichments and microcosms using metagenomic sequences. First, sequences from soil bacterial enrichments and soil microcosms were coassembled in order to generate metagenome-assembled genomes (MAGs) and associate possible resistome elements to concrete taxa. Metagenomic reads obtained from soil microcosms or bacterial enrichments were filtered according to the criteria described by Minoche et al. [49]. Then, reads were coassembled using MEGAHIT [50] to generate contigs, and reads were mapped onto the contigs using Bowtie 2 [51] to generate BAM files. Profiles were created for each individual sample and merged using the anvi’o metagenomic workflow [52]. Samples were binned based on differential coverage and sequence composition. A bin was considered as a MAG when it showed a completion higher than 50% and a redundancy lower than 10%. In order to determine whether the assembled contigs contained ARGs, the merged profile was blasted against the CARD database [53] using Diamond. The obtained results were filtered at a minimum identity of 60% and a minimum length of 33 amino acids, and the best hit was selected. A summary of these assemblies is found in Table S3. Since the assembly represented a very low proportion of the total of sequenced reads from soil microcosms, information regarding soil resistome composition could be lost using this approach. Therefore, the resistome screening was carried out on nonassembled reads from bacterial enrichments and soil microcosms in order to evaluate all the sequences. Reads were trimmed using the Fastq Quality Trimmer tool of the FASTX-Toolkit. Nucleotides that did not meet a minimum quality score of Q20 were trimmed from the sequences, and sequences shorter than 100 nucleotides after trimming were removed. Then, reads from R1 and R2 were concatenated and blasted against the CARD database using Diamond. The obtained results were filtered at a minimum identity of 60% and a minimum length of 33 amino acids, and the best hit was chosen. Genes that had less than 10 copies in the ensemble of samples were removed, and the relative abundance of each gene was calculated. The genes that increased their relative abundance over time in nonpolluted samples were removed, i.e., the genes for which the Pearson coefficient between time and relative abundance in nonpolluted microcosms was higher or equal to 0.9. Finally, the genes that did not show a Pearson coefficient between time and relative abundance in polluted samples higher than 0.9 were also removed. The remaining genes were subjected to a leave-one-out cross-validation in order to determine whether their increase under gentamicin pollution over time was significant.

### 4.9. Gentamicin Bioavailability and Adsorption in Soil

One gram of unpolluted soil or soil polluted at 1 µg/g, 100 µg/g or 1 mg/g of gentamicin was diluted in 10 mL of water to determine the bioavailable fraction, then vortexed and stocked at −20 °C until analysis. Then, tubes were centrifugated at 10,000 rpm for 5 min to recover the bioavailable fraction of gentamicin. A 200 µL sample of supernatant was transferred to a polypropylene tube. Then, 200 µL of 75 mM sodium hexanesulfonate and 200 µL of 75 mM sodium heptanesulfonate were added to the tube, and after vortexing for 30 s, were analyzed by HPLC-MS/MS [54].

## Figures and Tables

**Table 1:** Antibiotic-resistance genes (ARGs) that increased their average relative abundance in soil enrichments in 1:10 TSB medium with different gentamicin concentrations after 24 h of incubation (Pearson coefficient > 0.9). Both genes showed a significantly higher abundance at 12 µg/mL of gentamicin compared to the rest of culture enrichments (*p*-values of 0.046 and 0.032 for *muxB* and *adeF*, respectively).

[Gentamicin]	0 µg/mL			0.1 µg/mL			12 µg/mL		
Sample/Gene	SC1	SC2	SC3	SC4	SC5	SC6	SC7	SC8	SC9
*muxB*	0	4.9 × 10^−6^	0	0	5.4 × 10^−6^	0	1.5 × 10^−4^	6.7 × 10^−5^	1.3 × 10^−4^
*adeF*	0	0	0	0	0	0	5.1 × 10^−5^	9.3 × 10^−5^	6.4 × 10^−5^

**Table 2:** Physical characterization of La Côte de Saint André soil (France).

Soil Parameter	La Côte de Saint André
Sand	42.9%
Silt	43.6%
Clay	13.5%
pH	7.24
Organic matter	2.92%
Organic C	1.7%
Total N	0.17%

## Data Availability

The datasets generated and analyzed during the current study are publicly available in the Environmental Microbial Genomics Group repository: ftp://ftp-adn.ec-lyon.fr/.

## Supplementary Materials

The following are available online at https://www.mdpi.com/2079-6382/10/2/191/s1, Figure S1. Bacterial community composition PCoA of: (**a**) soil microcosms and bacterial enrichments; (**b**) soil microcosms; (**c**) soil bacterial enrichments. Figure S2. Bacterial richness measured in soil microcosms and enriched media polluted at different gentamicin concentrations. * *p*-value ≤ 0.05; ** *p*-value ≤ 0.01; *** *p*-value ≤ 0.001; ***** p*-value ≤ 0.0001. *n* = 3; Table S1. Antibiotic-resistance genes that increased their average relative abundance over time in soil microcosms under gentamicin pollution. Table S2. Gentamicin concentrations in the available fraction of soils polluted at 1 µg/g, 100 µg/g and 1 mg/g. Table S3. Assembly and ARG screening on metagenomic reads obtained from soil bacterial enrichments (nonpolluted or polluted with gentamicin at 100 ng/mL or 12 µg mL) and from soil microcosms (nonpolluted or polluted with gentamicin at 1 µg/g, 100 µg/g or 1 mL/g).
