# Supplementary material for: Gentamicin Adsorption onto Soil Particles Prevents Overall Short-Term Effects on the Soil Microbiome and Resistome
Source: Antibiotics (Basel). 2021 Feb 15;10(2):191. doi: 10.3390/antibiotics10020191 (PMC7919497; doi:10.3390/antibiotics10020191)
Supplement: Supplementary file 1 [file antibiotics-10-00191-s001.pdf]

Supplementary Materials

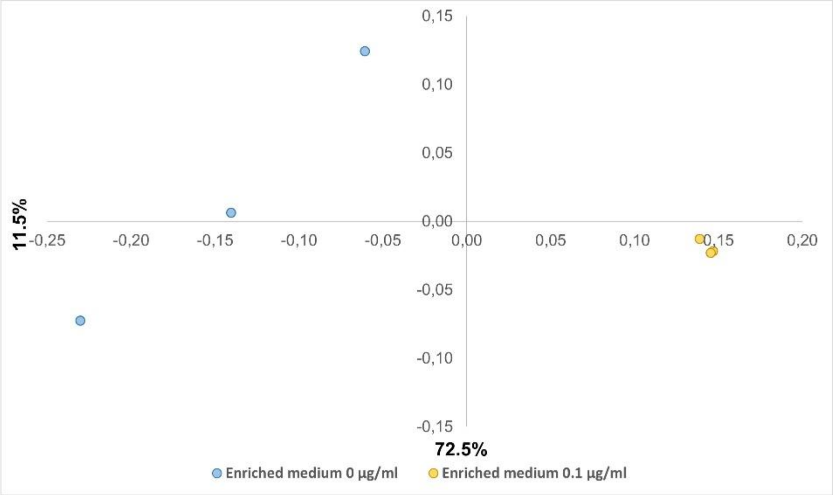

(a)

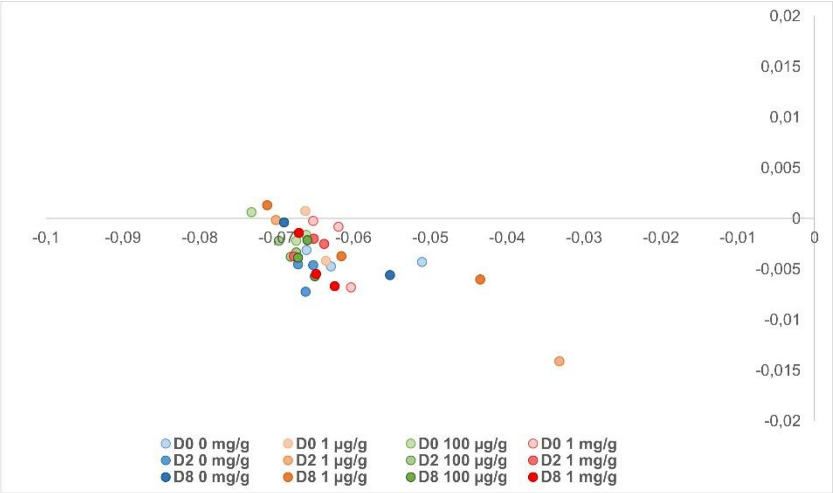

(b)

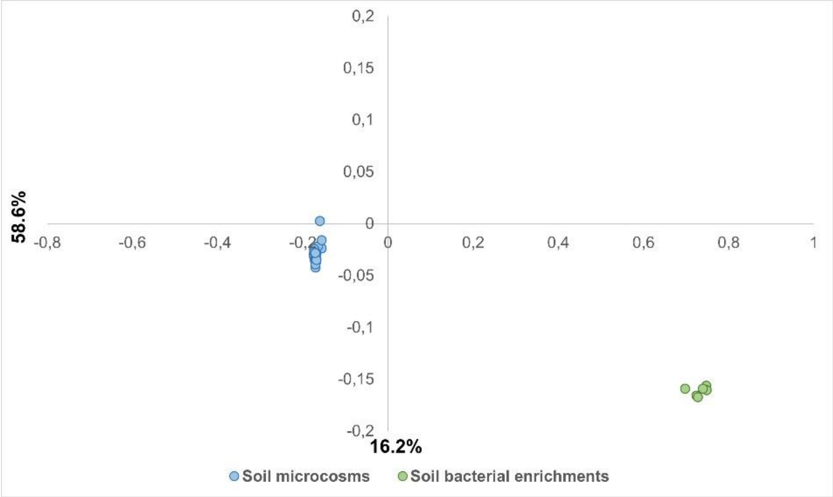

(c)

Figure S1. (a) Bacterial community composition PCoA of: (a) soil microcosms and bacterial enrichments; (b) soil microcosms; (c) soil bacterial enrichments.

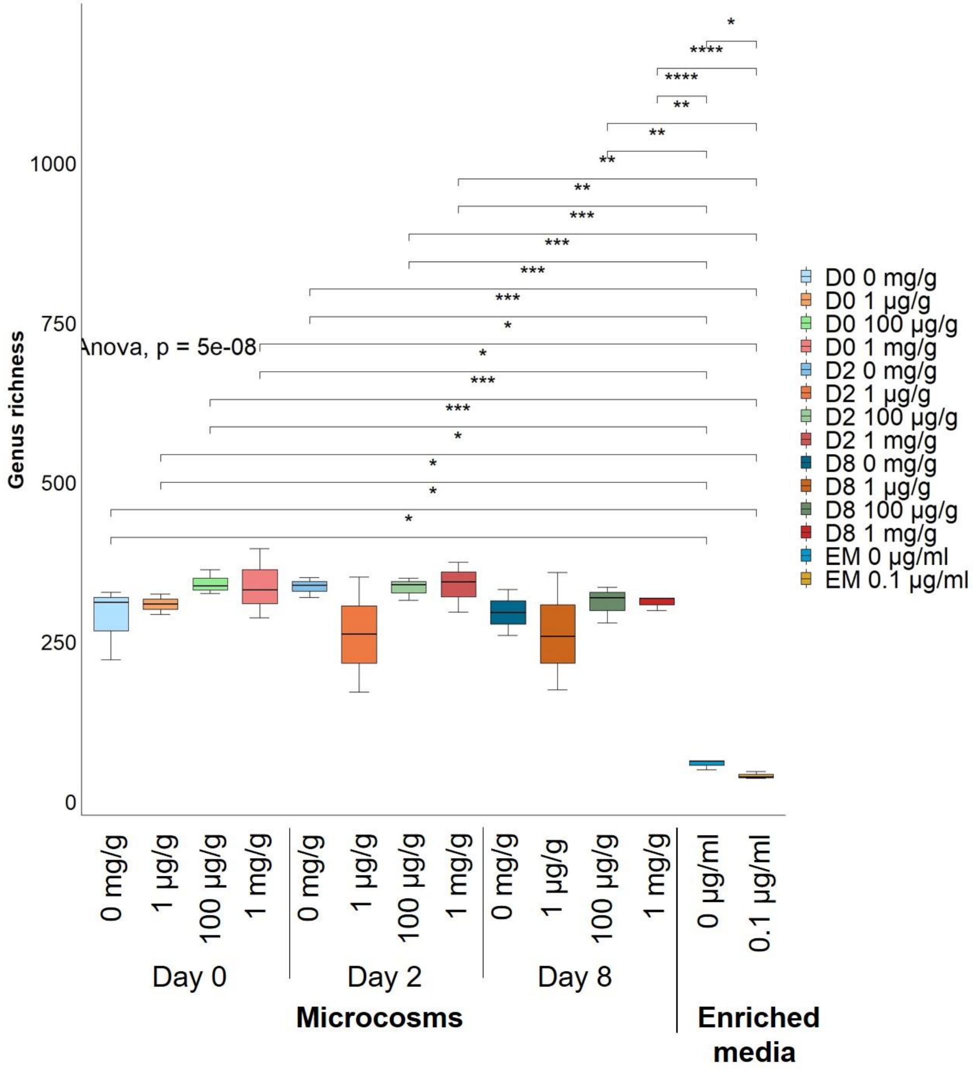

Figure S2. Bacterial richness measured in soil microcosms and enriched media polluted at different gentamicin concentrations. *p-value ≤ 0.05; **p-value ≤ 0.01; ***p-value ≤ 0.001; ***p-value ≤ 0.0001. n=3.

Table S1a. Genes related to aminoglycoside resistance that increased their average relative abundance over time in soil microcosms at different gentamicin (Pearson coefficient between time and gentamicin concentration ≥0.9). No significant increase over time in gentamicin-contaminated soils was detected for any of these genes. *ceoB* and *smeB:* multidrug efflux pumps that include aminoglycoside efflux. *acrD* and *amrB:* aminoglycoside efflux pumps. *baeR:* promoter of *acrD* expression. n=3.

	Day 0				Day 2				Day 8				Increased RA over time		
**ARG**	**0 µg/g**	**1 µg/g**	**100 µg/g**	**1 mg/g**	**0 µg/g**	**1 µg/g**	**100 µg/g**	**1 mg/g**	**0 µg/g**	**1 µg/g**	**100 µg/g**	**1 mg/g**	**1 µg/g**	**100 µg/g**	**1 mg/g**
ceoB	1,04E-05	0,00E+00	1,28E-05	7,53E-06	8,16E-07	6,48E-06	3,68E-06	7,84E-06	1,78E-06	1,83E-05	1,38E-05	1,30E-05	X		
acrD	0,00E+00	5,01E-06	0,00E+00	0,00E+00	2,75E-06	1,14E-06	2,16E-06	0,00E+00	0,00E+00	2,86E-06	8,79E-06	3,76E-06		X	
smeB	0,00E+00	2,96E-06	0,00E+00	2,80E-06	1,63E-06	0,00E+00	1,08E-06	0,00E+00	0,00E+00	2,86E-06	7,86E-06	1,44E-06		X	
amrB	3,91E-06	0,00E+00	0,00E+00	2,80E-06	0,00E+00	0,00E+00	1,52E-06	8,21E-07	0,00E+00	2,86E-06	4,19E-06	2,32E-06		X	
baeR	0,00E+00	0,00E+00	1,78E-06	1,40E-06	0,00E+00	1,14E-06	1,08E-06	0,00E+00	0,00E+00	2,86E-06	9,31E-07	2,32E-06	X		

Table S1b. Genes related to multidrug efflux that increased their average relative abundance over time in soil at different gentamicin (Pearson coefficient between time and gentamicin concentration ≥0.9). No significant increase over time in gentamicin-polluted soils was detected for any of these genes. n=3)

	Day 0				Day 2				Day 8				Increased RA over time		
**ARG**	**0 µg/g**	**1 µg/g**	**100 µg/g**	**1 mg/g**	**0 µg/g**	**1 µg/g**	**100 µg/g**	**1 mg/g**	**0 µg/g**	**1 µg/g**	**100 µg/g**	**1 mg/g**	**1 µg/g**	**100 µg/g**	**1 mg/g**
muxB	3,25E-05	4,20E-05	2,50E-05	4,85E-05	2,07E-05	3,50E-05	3,27E-05	3,28E-05	2,02E-05	3,34E-05	4,05E-05	2,55E-05		X	
mexF	2,08E-05	4,50E-06	9,46E-06	1,96E-05	1,27E-05	1,18E-05	6,27E-06	1,25E-05	3,56E-06	2,01E-05	2,21E-05	1,96E-05	X		
msbA	2,08E-05	9,09E-06	1,04E-05	2,40E-05	4,38E-06	9,93E-06	9,46E-06	8,74E-06	2,05E-05	1,02E-05	2,19E-05	2,28E-05	X		
mexQ	3,91E-06	0,00E+00	9,36E-06	8,58E-06	6,43E-06	1,91E-06	5,40E-06	1,62E-06	0,00E+00	2,11E-05	1,35E-05	9,18E-06	X		
mdsB	8,53E-06	5,01E-06	0,00E+00	1,23E-05	1,63E-06	8,76E-06	2,60E-06	4,75E-06	6,92E-06	1,45E-05	1,90E-05	9,18E-06	X	X	
oqxB	3,91E-06	3,06E-06	1,78E-06	1,26E-05	5,32E-06	9,90E-06	5,83E-06	5,36E-06	5,13E-06	1,08E-05	1,09E-05	9,81E-06	X	X	
mexW	0,00E+00	6,03E-06	6,15E-06	1,40E-06	0,00E+00	9,16E-06	6,86E-06	4,69E-06	1,77E-05	1,23E-05	1,12E-05	3,10E-06	X	X	
acrB	0,00E+00	0,00E+00	0,00E+00	7,02E-06	1,93E-06	4,20E-06	1,08E-06	4,56E-06	0,00E+00	2,86E-06	8,59E-06	2,32E-06		X	
acrF	0,00E+00	0,00E+00	0,00E+00	6,13E-06	1,63E-06	4,20E-06	1,08E-06	1,60E-06	0,00E+00	4,30E-06	5,12E-06	1,55E-06		X	
mtrA	3,91E-06	0,00E+00	0,00E+00	3,33E-06	8,16E-07	3,42E-06	0,00E+00	1,62E-06	5,13E-06	5,12E-06	2,79E-06	6,19E-06	X		
rosA	5,21E-06	1,53E-06	1,78E-06	0,00E+00	0,00E+00	5,71E-06	0,00E+00	1,62E-06	0,00E+00	4,51E-06	2,79E-06	2,32E-06			X

Table S1c. Genes related to tetracycline efflux that increased their average relative abundance over time in soil microcosms at different gentamicin concentrations (Pearson coefficient between time and gentamicin concentration ≥0.9). No significant increase over time in gentamicin-polluted soils was detected for any of these genes. N=3.

ARG	Day 0				Day 2				Day 8				Increased RA over time		
	0 µg/g	1 µg/g	100 µg/g	1 mg/g	0 µg/g	1 µg/g	100 µg/g	1 mg/g	0 µg/g	1 µg/g	100 µg/g	1 mg/g	1 µg/g	100 µg/g	1 mg/g
tetB(60)	0,00E+00	0,00E+00	1,78E-06	1,26E-05	2,75E-06	3,05E-06	1,08E-06	3,11E-06	0,00E+00	8,59E-06	4,19E-06	8,15E-06	X		
tetA(58)	3,91E-06	5,93E-06	1,78E-06	1,40E-06	0,00E+00	6,48E-06	3,97E-06	4,58E-06	0,00E+00	7,21E-06	6,38E-06	6,08E-06	X	X	X
otrC	5,21E-06	0,00E+00	0,00E+00	0,00E+00	8,16E-07	4,20E-06	1,08E-06	8,21E-07	0,00E+00	1,65E-06	1,40E-06	3,76E-06		X	X
otr(A)	0,00E+00	0,00E+00	2,59E-06	5,61E-06	0,00E+00	1,91E-06	3,97E-06	8,21E-07	0,00E+00	3,69E-06	1,77E-06	2,18E-06	X		
adeB	0,00E+00	0,00E+00	0,00E+00	0,00E+00	0,00E+00	1,91E-06	0,00E+00	0,00E+00	0,00E+00	2,86E-06	6,63E-06	0,00E+00	X		

Table S1d. Genes related to other resistance mechanisms that increased their average relative abundance over time in soil microcosms under gentamicin pollution (Pearson coefficient between time and gentamicin concentration ≥0.9). No significant increase over time in gentamicin-polluted soils was detected for any of these genes. *Streptomyces:* mutant conferring resistance to aminocoumarin. *rphA:* rifampicin enzymatic inactivation. *BIfidobacteria:* intrinsically resistant form of *ileS* (isoleucyl-tRNA synthetase) in *Bifidobacteria* that confers resistance to mupirocin. *mupA:* alternative *ileS* in *Staphylococcus* conferring resistance to mupirocin. *bcrA:* ABC transporter conferring resistance to bacitracin. n=3.

ARG	Day 0				Day 2				Day 8				Increased RA over time		
	0 µg/g	1 µg/g	100 µg/g	1 mg/g	0 µg/g	1 µg/g	100 µg/g	1 mg/g	0 µg/g	1 µg/g	100 µg/g	1 mg/g	1 µg/g	100 µg/g	1 mg/g
Streptomyces	0,00E+00	2,28E-05	3,56E-06	7,18E-06	1,63E-06	6,48E-06	5,05E-06	7,16E-06	0,00E+00	1,11E-05	7,86E-06	7,52E-06		X	
rphA	3,91E-06	0,00E+00	4,27E-06	1,93E-06	2,75E-06	1,14E-06	5,05E-06	7,98E-06	5,13E-06	6,61E-06	6,38E-06	4,95E-06	X	X	
Bifidobacteria	0,00E+00	1,53E-06	5,33E-06	3,33E-06	8,16E-07	1,14E-06	0,00E+00	3,89E-06	0,00E+00	3,11E-06	7,31E-06	4,32E-06			X
mupA	0,00E+00	0,00E+00	0,00E+00	1,93E-06	1,93E-06	1,14E-06	0,00E+00	8,02E-07	0,00E+00	2,89E-06	3,00E-06	3,76E-06	X		
bcrA	0,00E+00	5,01E-06	0,00E+00	0,00E+00	8,16E-07	1,14E-06	1,08E-06	8,02E-07	0,00E+00	2,86E-06	1,40E-06	2,18E-06		X	X

Table S2. Gentamicin concentrations in the available fraction (in the water fraction) of soils polluted at 1 µg/g, 100 µg/g and 1 mg/g. <QL= below quantification limit.

Gentamicin concentration	Bioavailable fraction
0 ng/g	**<QL**
1 µg/g	**<QL**
100 µg/g	**<QL**
1 mg/g	**<QL**

Table S3. Assembly and ARG screening of metagenomic reads obtained from soil bacterial enrichments (at different gentamicin concentrations: 0, 0.1 µg/ml or 12 µg ml) and from soil microcosms (at different gentamicin concentrations: 0, 1 µg/g, 100 µg/g or 1 ml/g).

		Soil bacterial enrichments	Soil microcosms
Co-assembly of metagenomic reads (MEGAHIT)	Input	2908668 reads (9 samples)	9556334 reads (36 samples)
	Assembled contigs	4155	137
	Contig length	1000 – 27273 bp	1000 – 2977 bp
	Average length	2238 bp	1179 bp
	N50	2417 bp	1147 bp
Mapping (Bowtie2)	Maximum alignment rate	85.47%	0.08%
Binning	Bins wih >50% completion and <10% redundancy	2 (*Bacillus* and *Lysinibacillus)*	0
ARG screening on anvi'o profiles	Hits with >60%id and >33 aa	38 hits	0 hits
